# The relationships between social internet use, social contact, and loneliness in older adults

**DOI:** 10.1038/s41598-025-09861-8

**Published:** 2025-07-12

**Authors:** Jeroen H.M. Janssen, Theo G. van Tilburg, Erik J. van Ingen, Rense Corten, Geeske Peeters, Marcel G.M. Olde Rikkert

**Affiliations:** 1 Department of Geriatric Medicine, Radboud University Medical Center, Nijmegen, The Netherlands; 2 Department of Developmental Psychology, Tilburg University, PO Box 90153, Tilburg, 5000 LE The Netherlands; 3 Department of Sociology, Vrije Universiteit Amsterdam, Amsterdam, The Netherlands; 4 Department of Sociology/ICS, Utrecht University, Utrecht, The Netherlands; 5 Radboudumc Alzheimer Center, Radboud University Medical Center, Nijmegen, The Netherlands; 6 Department of Geriatric Medicine, Donders Institute for Brain, Cognition and Behaviour, Radboud University Medical Center, Nijmegen, The Netherlands

**Keywords:** Longitudinal mediation, Social connectedness, Social media, Contact frequency, Human behaviour, Quality of life

## Abstract

Social internet use might decrease loneliness, potentially by increasing social contact. Vice versa, loneliness might decrease social internet use by decreased social contact. However, few studies explored these associations longitudinally. This study aimed to assess the longitudinal, bidirectional associations between frequency of social internet use, loneliness, and social contact (measured as the number of frequently contacted personal network members). We used four waves of the Longitudinal Aging Study Amsterdam (2011-13, 2015-16, 2018-19, 2021-22) in a sample of 1923 Dutch older adults (age 55–98). We applied random intercept cross-lagged panel models to test the bidirectional and mediational relationships. We found a longitudinal association between social internet use and subsequent loneliness, β = -0.07, *p* = .008, but no evidence of mediation of social contact, nor of a reversed association. These results strengthen existing evidence for a positive association of social internet use in decreasing loneliness. In contrast, more loneliness did not predict a difference in social internet use, suggesting that this reversed relationship is more complex or dependent on individual characteristics.

## Introduction

Due to population ageing, the number of older adults experiencing loneliness is expected to increase in the coming decades^1^. Persistent loneliness has been associated with severe health consequences, including an increased risk of cognitive impairment, dementia progression, hypertension, heart and coronary diseases, and all-cause mortality^2–4^. Older adults are especially at risk of experiencing loneliness due to losing close contacts and declining physical, mental, and cognitive health and mobility (for an overview of longitudinal risk factors, see Dahlberg et al.^5^). These factors limit the resources needed for sustained in-person contact, thereby complicating relationship maintenance^6,7^.

Digital contact, such as social internet use, can provide interaction opportunities despite geographical distance or mobility decline. This allows maintaining a larger network with contacts that would otherwise potentially be lost, and helps prevent loneliness. In this study, we explore the bidirectional, longitudinal relationships between social internet use and loneliness. Furthermore, we propose mediation of increased social contact as a potential mechanism underlying these associations.

### How social internet use affects loneliness

Internet use plays an increasingly large part in older adults’ (social) lives. From 2013 to 2023, daily internet use among Dutch adults aged 65–75 increased from 56 to 91%; people aged 75 and older increased from 20 to 66% in the same period. One of the most important online activities is social internet use (SIU), which entails a large variety of activities, including but not limited to, social media, e-mail, instant messaging, or video calling. In 2023, 93% of people aged 65–75 and 74% of people aged 75 and older used the internet for instant messaging^8^.

Older adults can benefit from SIU in a variety of ways, providing advantages above mere face-to-face contact^9^. Social media, for instance, makes it easier to stay updated with a large network of both family and friends and weaker ties. Furthermore, SIU offers additional interaction opportunities complementing in-person contact with family and friends. This allows for interactions despite mobility decline and geographical distance, and facilitates more frequent contact with larger groups of strong ties^10,11^.

Through these facilitating mechanisms, SIU might affect loneliness, though results on this are still mixed. While some cross-sectional studies found that more SIU is associated with lower loneliness^12–15^recent reviews are inconclusive^16–18^. This is partly attributed to a lack of longitudinal studies preventing identification of underlying causal mechanisms. The two longitudinal studies on this topic strengthen the evidence that SIU might decrease loneliness. The study of Szabo et al.^19^ found in a sample of New Zealand adults aged 55–70 that more frequent SIU predicted lower loneliness over three waves, although SIU was only assessed at the first wave, preventing temporal ordering of variables. Furthermore, Yu et al.^20^ found similar results with a generalized measure of internet use, only assessing whether respondents regularly use the internet.

These two studies also exemplify the second reason for mixed results in previous reviews: the lack of sensitive measures of SIU. Without longitudinal measures including specific online activities and duration of use, identifying specific mechanisms is difficult^21,22^. This is important, as it is hypothesized that internet use only may decrease loneliness when used for social interaction^23^. Therefore, the first objective of this study is to provide evidence on the possible effect of the frequency of SIU on loneliness using longitudinal data collected in a large community-based population of older adults. Based on previous longitudinal studies^19,20^we hypothesize:

H1. More frequent social internet use over time is associated with lower loneliness in older adults.

#### Mediation of social contact

One of the pathways through which SIU can decrease loneliness is by facilitating contact. While SIU also allows for weak-tie formation and maintenance through social media, this article focuses on contact with personal network members. This network comprises those ties with whom an individual has frequent contact and who are important to them. Previous cross-sectional studies have already established a positive link between SIU and social contact^13,24,25^which in turn is an important predictor for loneliness^26^. SIU provides opportunities for increased interaction with existing ties^27^allows reconnecting with latent ties, and facilitates the formation of new ties^28,29^. Older adults use SIU to compensate for the lack of in-person interaction possibilities due to mobility issues^30^. A recent longitudinal study has shown that more frequent SIU is associated with both personal network stability and growth^31^.

To our knowledge, only two longitudinal studies have specifically investigated this mediation model. Yu et al.^20^ utilized data from three waves of the Health and Retirement Study with a single, dichotomous item to measure internet use, asking participants if they regularly use the internet. This measure did not differentiate between social and non-social use and did not incorporate frequency of use. Zhang et al.^32^ used a more precise measure of SIU, which measured the time spent on social media for communicating with family or friends. They used two waves of the Health and Retirement Study, with all measures except loneliness solely administered at the second wave. This makes it impossible to draw conclusions about the temporal direction of the association. The findings supported partial mediation, although methodological considerations prevented strong conclusions. Regarding this mediation model, we therefore hypothesize that

H2. More frequent social internet use in older adults is (a) positively associated with social contact, which in turn is (b) negatively associated with loneliness.

### How loneliness affects social internet use

In addition to SIU potentially affecting loneliness in older adults, it is proposed that the reversed relationship also exists, i.e., loneliness affecting SIU^23^. However, the literature provides no clear conclusion regarding the direction of this relationship^33^. One vein of research comes from the opposing *social compensation* and *social enhancement* hypotheses. The social compensation (or poor-get-richer) hypothesis posits that lonely individuals, who are known to socially withdraw and avoid social rejection^34^might benefit more from online interactions, as they are more asynchronous and allow for better control over the situation^33^. According to this hypothesis, lonely individuals could turn to and improve from SIU. One study found that older adults rely on Facebook to compensate for the lack of in-person social contact^35^.

On the other hand, the social enhancement (or rich-get-richer) hypothesis^36^ states that individuals with the strongest network are most likely to benefit from SIU, as they have the most online possibilities to increase their social resources^33,37^. This could mean that lonely older adults are less likely to increase SIU. For older adults, one of the main reasons for using Facebook is to stay up-to-date on family and friends^30^. If there are fewer contacts available to interact with online, SIU might be less preferred.

This latter reasoning links to research on technology adoption. It is known that older adults are more inclined to use digital technology if there is existing support from the network, primarily from strong tie family and friends^38^. Adoption of SIU is more likely after others’ recommendations or through observational learning^39^. Furthermore, these ‘local experts’ also provide support throughout the adoption process^40,41^.

Following the argumentation from technology adoption studies and the social enhancement hypothesis, a mediation model between SIU, loneliness and social contact with reversed causal order holds. Lonely individuals are less likely to use SIU, because they have a smaller network and fewer available people to invite or support them, or fewer available people to interact with online. We therefore hypothesize that

H3. Higher loneliness is negatively associated with frequency of SIU.

H4. Higher loneliness is (a) negatively associated with social contact, which is (b) positively associated with frequency of SIU.

**Fig. 1:**
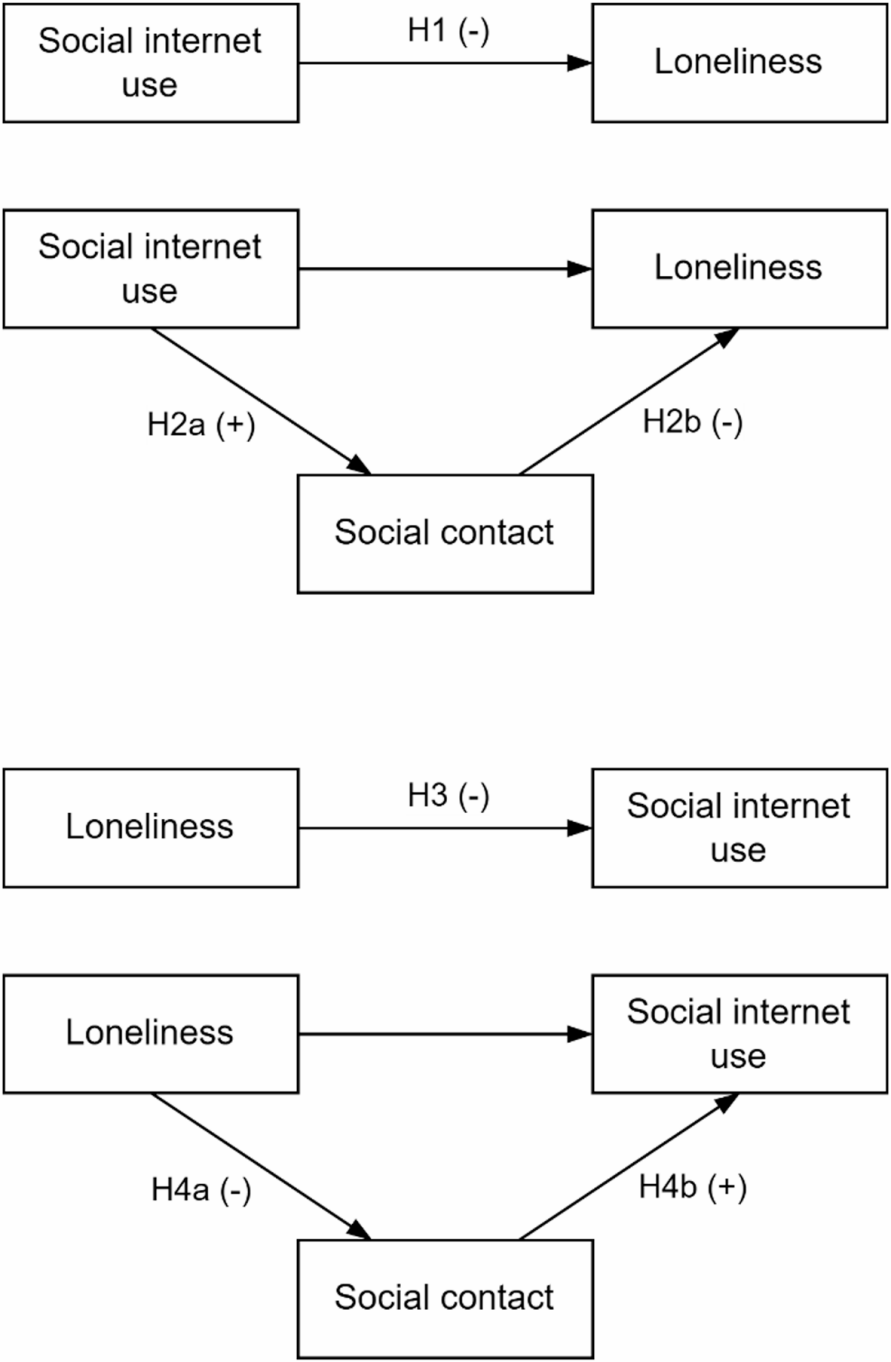
Conceptual models of loneliness, social internet use, and social contact, with annotated hypotheses.

Figure 1 shows the conceptual model with all proposed hypotheses. These hypothesized pathways form a vicious cycle: people higher in loneliness use less SIU, which further increases loneliness. This vicious cycle has previously been described, stating that loneliness hinders the ability to maintain relationships, which further increases loneliness^7^. Longitudinal research found reciprocal relationships between loneliness and social integration^42^. We propose a similar cycle between SIU and loneliness, through mediation of social contact.

## Methods

### Participants

We used data from the *Longitudinal Aging Study Amsterdam* (LASA), an ongoing longitudinal cohort study in the Netherlands^43^. The LASA study is conducted in accordance with the Declaration of Helsinki. Ethical approval was provided by the ethical committee of the VU Medical Center. Participants signed informed consent prior to data collection.

LASA collects data on physical, cognitive, emotional, and social functioning. Data collection consists of three steps, all performed at the respondents’ home: (1) a voice-recorded main interview, administered by a trained interviewer, taking approximately 2 h to complete; (2) a self-administered questionnaire, to be filled in on paper or online in respondents’ own time and pace; and (3) in case of consent, a subsequent medical interview including clinical measurements, taking approximately 1.5 h. Respondents who refused to participate in the main interview because they were too frail or busy were offered an abbreviated 15-minute telephone interview, covering a selection of key variables^43^. We did not include telephone interviews, as these did not include data on social networks or SIU.

LASA started in 1992 with a cohort of adults aged 55–84 from three regions in the Netherlands (Cohort 1, *n* = 3107). Two additional cohorts were included from the same sampling frame, Cohort 2 in 2002 (*n* = 1002) and Cohort 3 in 2012 (*n* = 1023). Measurements are taken every three to four years, with currently eleven waves available. We used data collected on waves 2011-13 (T1, *n* = 2545, including the baseline observation of Cohort 3), 2015-16 (T2, *n* = 2024), 2018-19 (T3, *n* = 1701), and 2021-22 (T4, *n* = 1393). We excluded the 521 respondents who only participated in T1 and the 101 participants with missing values on all independent and dependent variables. Our analytical sample consisted of *N* = 1923 participants, with a mean age of 67.8 (Standard Deviation = 8.7) of whom 54% were female. Compared to our analytic sample, the 622 excluded participants were older, more often single, and had fewer years of education.

### Measures

#### Social internet use

For SIU, respondents were first asked the question, (1) “Do you use the Internet? You can use the Internet on, for example, a computer, tablet, smartphone, or smart TV.” If answered *yes*, respondents were asked to select from a list the online activities they do, one of which is (2f) *Maintaining contact with family*,* friends*,* or neighbors (for example*,* via email*,* WhatsApp*,* Skype*,* and social media like Facebook).* If this activity is selected, they received the question, (3) “How often do you use the Internet for contact with family, friends, or neighbors?” with five response categories, which were recoded into a score reflecting the number of SIU days per year: *less than a few times per year* = 3, *a few times per year* = 10, *a few times per month* = 60, *a few times per week* = 156, and *daily* = 365. We assigned *never* = 0 to participants who answered *no* to question (1) or did not select function (2f) and thus did not answer question (3).

#### Social contact

LASA uses the domain-contact method to measure a respondent’s personal network^44^. This method uses name-generator questions to identify network members important to participants and with whom they have regular contact, divided into seven domains. The interviewer then asked, “I would like to know how often you have contact with the persons I am about to mention. This includes contact both when you see each other and when you call or write to each other.” For every alter, response categories were: *never* = 0, *yearly or less often* = 1, *a few times per year* = 2, *monthly* = 3, *once every two weeks* = 4, *weekly* = 5, *a few times per week* = 6, and *daily or household member* = 7. Per respondent, we counted the number of relationships with whom the respondent had at least weekly contact (i.e., answered 5 or higher).

#### Loneliness

LASA measured loneliness with the De Jong Gierveld Loneliness Scale^45^. This scale contains 11 statements, both positively and negatively formulated, that respondents answered with *no* = 1, *more or less* = 2, or *yes* = 3. The item scores are dichotomized – that is, recoded as *no* = 0, *more or less/yes* = 1 for positive items, and *yes* = 0, *more or less/no* = 1 for negative items – and summed to receive the loneliness score (range 0–11, higher scores meaning higher loneliness). A score of 0–2 indicates no, 3–8 moderate, and 9–11 severe loneliness^46^. The scale score was set to missing if more than one of the item scores were missing. The scale validly measures overall loneliness^45,47^. In our sample, Cronbach’s α ranged from 0.83 to 0.84 across waves, indicating good internal consistency.

#### Control variables

We controlled for sex (male/female), partner status (yes/no), work status (the total number of paid and voluntary hours of work per day), and health status. For health status, respondents indicated for seven activities of daily living (ADL) how easily they could perform them (‘without help,’ ‘with some difficulty,’ ‘with much difficulty,’ ‘only with help,’ or ‘cannot perform’). The variable counts the number of ADL indicated as ‘with some difficulty’ or worse (range 0–7).

Regressing the time-specific within-person latent variables on the time-specific covariates caused estimation issues, resulting in model non-convergence. This is probably due to the large number of parameters needed to be estimated. Therefore, to ensure model convergence, we regressed the within-person latent variables on the covariance values of T1, while allowing the parameters to be freely estimated across time^48^.

### Statistical analyses

The data were analyzed using random-intercept cross-lagged panel models (RI-CLPM)^49^. Cross-lagged models are suitable for longitudinal research when the interest is in two or more variables’ influence on each other over time^49^. They allow to test the plausibility of different pathways, which is why they are suitable for this study. The RI-CLPM extends the classic cross-lagged panel model (CLPM) by including a random intercept that captures time-invariant, stable between-person differences. As a result, the cross-lagged coefficients in the RI-CLPM reflect the true within-person variability. As these two sources of variation are conflated in CLPM, the cross-lagged coefficients may be smaller or even non-significant in RI-CLPM compared to CLPM if some of the within-person variation is trait-like, while the fit of the RI-CLPM is often better^48,49^. This is especially relevant for loneliness, which is known to be at least partially trait-like^50^.

**Fig. 2:**
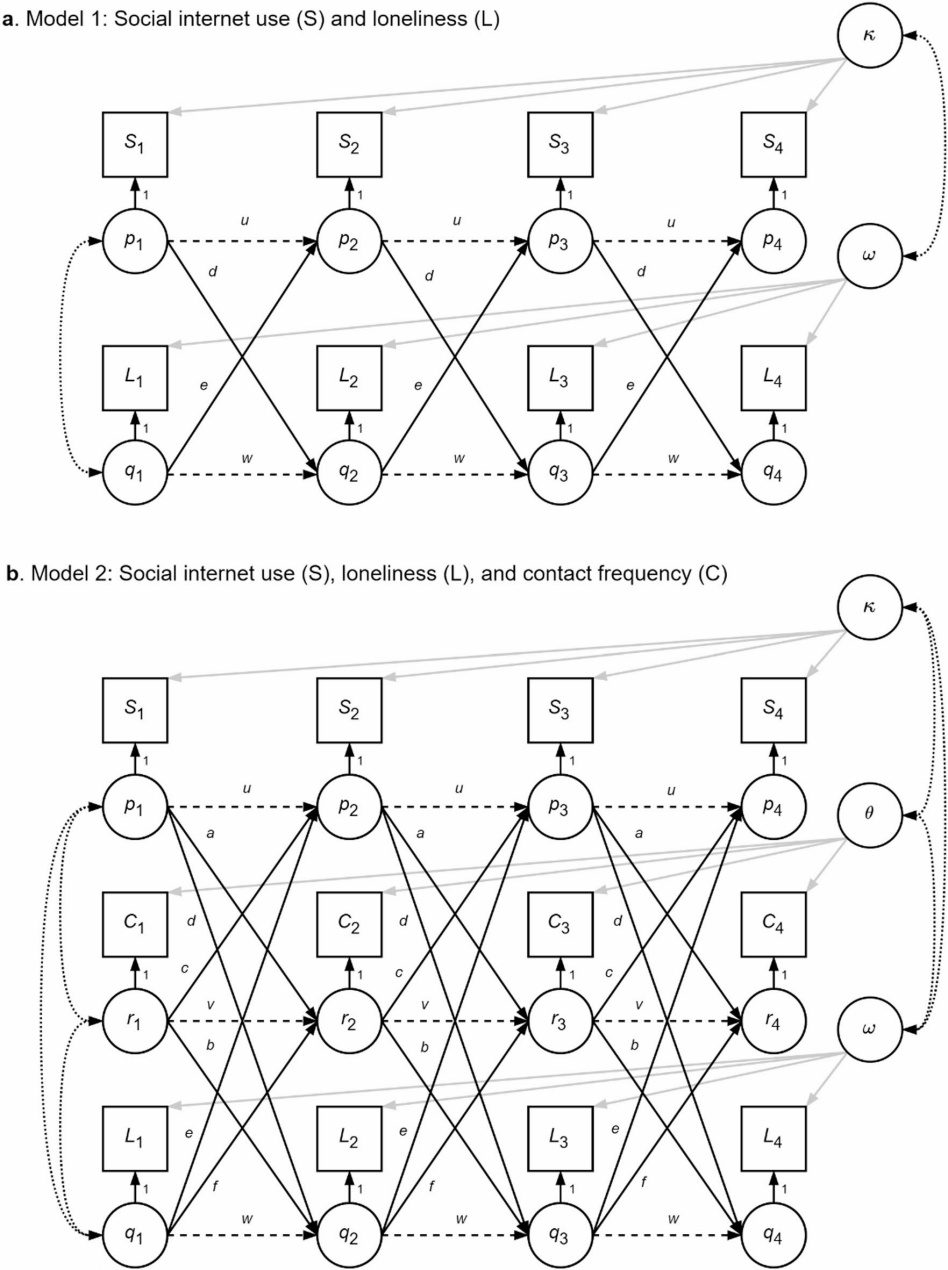
The RI-CLPM models. (**a**) The RI-CLPM with social internet use and loneliness; (**b**) The RI-CLPM with social internet use, social contact, and loneliness. Squares indicate observed variables; circles indicate latent variables. Variable subscripts 1, …, 4 denote timepoints. *S* = Social internet use; *L* = Loneliness; *C* = Social contact; Cov = Covariates. *p*, *q*, and *r *are the individual time-specific deviations from expected scores; κ, ω, and θ the random intercepts. Solid black lines represent cross-lagged effects (*a*,* b*,* c*,* d*,* e*, and *f*), dashed lines are autoregressive effects (*u*, *w*, and *v*), grey solid lines the effects of the random intercept, and grey dotted lines are covariances. Means and errors omitted.

Figure 2 shows a graphical representation of the RI-CLPMs. To test H1 and H3, we used Model 1, a RI-CLPM of SIU and loneliness. To address H2 and H4, we expanded Model 1 by adding social contact as a third variable (Model 2). In both models, all main and control variables are specified as observed indicators. The observed indicators are decomposed in a latent random intercept (κ, ω, and θ) and a within-person score (*p*, *q*, and *r*), reflecting the within-person deviation from an individual’s expected score. The autoregressive pathways between the within-person scores (*u*, *v*, and *w*) reflect within-person carry-over effects, while the cross-lagged pathways (*a*,* b*,* c*,* d*,* e*, and *f*) represent spill-over effects between variables^48^.

We used full information maximum likelihood (FIML) estimation with robust standard errors and a scaled test statistic, to account for missing data and binary covariates^51^. We assessed model fit using the χ^2^ test and the following fit statistics and cut-off values^52^: Root Mean Square Error of Approximation (RMSEA) ≤ 0.06, Standardized Root Mean Square Residual (SRMR) ≤ 0.08, and Comparative Fit Index (CFI) ≥ 0.90. Nested models were compared using model fit along with Akaike’s Information Criterion (AIC), Bayesian Information Criterion (BIC), and the chi-squared difference test (Δχ^2^). The Δχ^2^ test statistically tests the difference between two models against a χ^2^-distribution, although it is sensitive to sample size. The CLPM is essentially a RI-CLPM with the (co-)variances of the random intercept fixed to zero, but the constraints occur on the bound of the parameter space. Therefore, a regular Δχ^2^ test is too conservative; instead, we used the Δ$\bar{\chi}$^2^ test^48^.

#### Equality constraints

A RI-CLPM with freely estimated parameters was initially estimated. To test whether the regression parameters and grand means were time-invariant (for parsimony and interpretability), we compared the model fit of the freely estimated and constrained model. Given the lower BIC value for the constrained model, while still retaining good model fit, we used the constrained model in the subsequent analyses. See Fig. 2 for the final model. Model fit for all models is given in Supplementary Table 1. For comparison, we fitted both a RI-CLPM and a regular CLPM, but this model fitted substantially worse than the RI-CLPM.

#### Meditation effects

For Model 2, we assessed mediation by calculating the overall direct effect (ODE), overall indirect effect (OIE), and overall total effect (OTE)^53^. The ODE is the sum of all pathways from X at T1 to Y at T4 without passing through M, where a pathway is defined as the product of the concerned path coefficients. For the relationships between SIU and loneliness, the ODE is defined as:

$$\:\begin{array}{c}OD{E}_{SIU\to Lon}=u\cdot u\cdot d+u\cdot d\cdot w+d\cdot w\cdot w+d\cdot e\cdot d\\\:OD{E}_{Lon\to SIU}=w\cdot w\cdot e+w\cdot e\cdot u+e\cdot u\cdot u+e\cdot u\cdot e\end{array}$$

The *OIE* is the sum of all effects from X at T1 to Y at T4, while passing through M at time *i*, with 1 < *i* < 4^53^. This is defined as:

$$\:\begin{array}{c}OI{E}_{SIU\to Lon}=u\cdot a\cdot b+a\cdot v\cdot b+a\cdot b\cdot w+d\cdot f\cdot b+a\cdot c\cdot d\\\:OI{E}_{Lon\to SIU}=w\cdot f\cdot c+f\cdot v\cdot c+f\cdot c\cdot u+e\cdot a\cdot c+f\cdot b\cdot e\end{array}$$

Lastly, the *OTE* is the sum of the *ODE* and *OIE*.

#### Robustness checks

The variable *social contact* counts the number of ties with whom the respondent has at least weekly contact. We also fitted the final model retained for Model 2 using different cut-off values (i.e., contact at least a few times per week, two-weekly, or monthly). This did not change the final conclusions (Supplementary Table 2).

#### Post-hoc analysis

To get a more in-depth understanding of the underlying mechanisms, we have fitted Model 2 with contemporaneous (lag0) effects rather than cross-lagged effects as post-hoc analysis. This did not change the final conclusions (Supplementary Table 3).

## Results

### Sample description

Of the 1923 respondents at T1, 1431 (74%) had a partner. The average number of ADLs performed with at least some difficulties was 1.1 (*SD* = 1.7). The average number of hours worked per week was 11.2 (*SD* = 16.1). At T1, 75% reported no feelings of loneliness, 22% reported moderate loneliness, and 3% reported severe loneliness. At T4, these numbers changed to 69%, 27%, and 4%, respectively. The frequency of SIU increased over time. While at T1, 34% reported no SIU, and 17% reported daily use, at T4, 18% reported no SIU, and 44% engaged in daily SIU. The average number of ties with whom the older adults had weekly contact or more often was 9.3 on T1 (*SD* = 5.6) and 8.0 on T4 (*SD* = 4.8). The correlation matrix (Table 1) shows negative, small to medium bivariate associations between SIU and loneliness, as well as between social contact and loneliness, while correlations between SIU and social contact are small to medium and positive.

**Table 1:** Sample statistics and bivariate correlation coefficients between social internet use, loneliness, and social contact at the different timepoints.

					Correlation coefficients										
Variable	Range	*N*	*M*	*SD*	1	2	3	4	5	6	7	8	9	10	11
1. Social internet use T1	0–365	1921	114.7	130.0											
2. Social internet use T2	0–365	1748	136.4	142.1	0.53										
3. Social internet use T3	0–365	1381	168.5	149.0	0.44	0.54									
4. Social internet use T4	0–365	1103	205.9	149.7	0.41	0.48	0.59								
5. Loneliness T1	0–11	1919	1.7	2.4	−0.09	−0.12	−0.13	−0.15							
6. Loneliness T2	0–11	1737	1.8	2.4	−0.11	−0.16	−0.16	−0.20	0.70						
7. Loneliness T3	0–11	1380	1.8	2.5	−0.11	−0.18	−0.20	−0.21	0.67	0.75					
8. Loneliness T4	0–11	1102	2.0	2.6	−0.10	−0.14	−0.20	−0.22	0.63	0.67	0.74				
9. Social contact T1	0–45	1865	9.3	5.6	0.16	0.16	0.20	0.23	−0.28	−0.27	−0.25	−0.22			
10. Social contact T2	0–45	1649	8.9	5.6	0.14	0.23	0.25	0.26	−0.27	−0.32	−0.28	−0.26	0.65		
11. Social contact T3	0–45	1326	8.3	5.1	0.12	0.14	0.23	0.22	−0.26	−0.28	−0.30	−0.29	0.61	0.66	
12. Social contact T4	0–45	1044	8.0	4.8	0.12	0.13	0.22	0.29	−0.27	−0.28	−0.26	−0.27	0.58	0.65	0.66

Correlations represent bivariate Pearson correlation coefficients, calculated using pairwise deletion. All correlations significant with *p* <.001. *M* = Mean, *SD* = Standard Deviation.

### RI-CLPMs

For both models, we retained the model with fixed regression parameters and fixed grand means. We found good model fit for both Model 1 (χ^2^(23) = 65.1, *p* =.562, RMSEA = 0.000, SRMR = 0.023, CFI = 0.99, AIC = 28406, BIC = 28700) and Model 2 (χ^2^(48) = 136.9, *p* <.001, RMSEA = 0.040, CFI = 0.99, SRMR = 0.025, AIC = 61695, BIC = 62195. For both models, the fit of the RI-CLPM was significantly better than the CLPM (Model 1: Δ$\bar{\chi}$^2^(3) = 203.0, *p* <.001; Model 2: Δ$\bar{\chi}$^2^(6) = 400.0, *p* <.001).

#### Does SIU predict loneliness through social contact (H1/H2)?

The results for both models are given in Table 2. Regarding the effect of SIU on loneliness, we found that more SIU predicted lower subsequent loneliness, *b* = −0.387, *p* =.008. This association remained significant when adding social contact to the model, *b* = −0.367, *p* =.011. This indicates that an increase in SIU is associated with a decrease in loneliness at a subsequent wave, which supports H1.

Regarding mediation of social contact, we found no significant associations between SIU and contact (H2a), *b* = −0.160, *p* =.601, or between contact and loneliness (H2b), *b* = −0.015, *p* =.169. The OIE was also not significant, *b* = −0.077, *p* =.650. This indicates that the association between SIU and loneliness is not mediated by social contact. We reject H2.

#### Does loneliness predict SIU through social contact (H3/H4)?

We also estimated the reversed prediction of loneliness on SIU, mediated by social contact. We found no significant association between loneliness and SIU, *b* = −0.012, *p* =.059. This effect remained non-significant when adding social contact to the model, *b* = −0.011, *p* =.081. Therefore, we find no evidence that higher loneliness predict less subsequent SIU, and we reject H3.

**Fig. 3:**
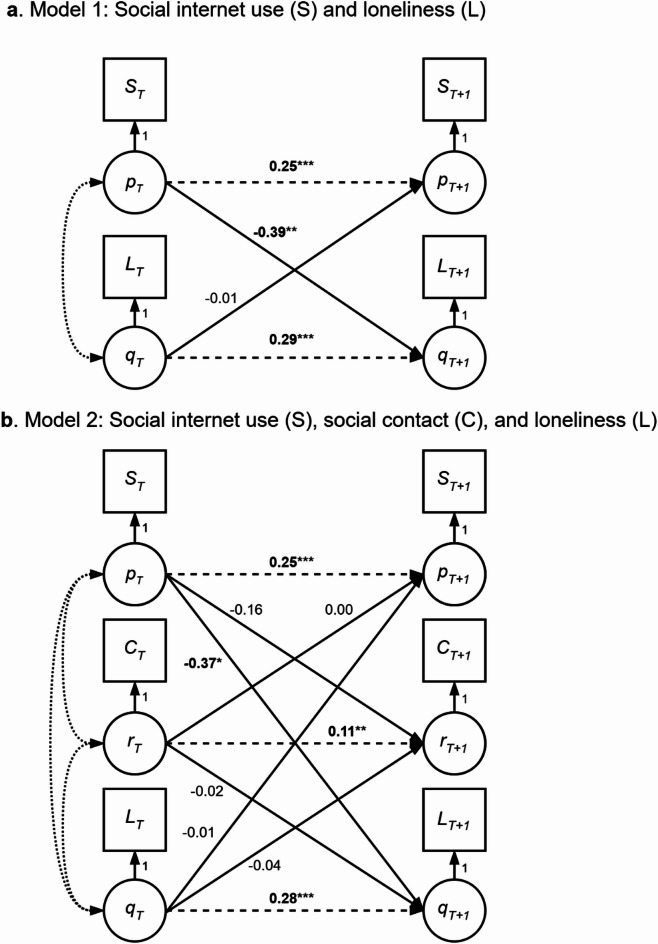
Model graph with unstandardized coefficients. As the coefficients are fixed over waves, they are the same for every combination of timepoint *T* and *T + 1*, with 1 < *T* < 3. * *p* < .05, ** *p* < .01, *** *p* < .001.

Regarding the mediation of social contact, we found no significant effect of loneliness on social contact (H4a), *b* = −0.038, *p* =.563, or of social contact on SIU (H4b), *b* = −0.015, *p* =.169. The OIE was also not significant, *b* = 0.000, *p* =.695. We reject H4. Figure 3 shows a graph of the unstandardized regression coefficients in the final model.

#### Post-hoc analysis

For Model 2, the mediating relationships with social contact are non-significant. Among other explanations, it could be that the underlying relationships have a contemporaneous rather than lagged nature, meaning that the variables are associated at about the same timepoint. The RI-CLPM, assuming an effect three years later, then might not be able to detect these associations. To test this explanation, we fitted a new model, which is equivalent to the final Model 2, but models bidirectional lag0 effects instead of lag1 crossed effects. This model is statistically equivalent to Model 2 and thus has the same model fit. The conclusions of this model (see Supplementary Table 3) are the same as those of the RI-CLPM, showing only a significant relationship between SIU and loneliness, *b* = −1.570, *p* =.010.

**Table 2:** Parameter estimates for the final models retained for model 1 (social internet use and loneliness) and model 2 (social internet use, loneliness, and social contact).

		Model 1				Model 2			
	Effect	b/Cov	SE	95% CI	β/*r*	b/Cov	SE	95% CI	β/*r*
*Autoregressions*									
SIU	*u*	0.248***	0.033	[0.183, 0.314]	0.220	0.246***	0.033	[0.181, 0.311]	0.219
Loneliness	*w*	0.292***	0.068	[0.158, 0.426]	0.262	0.276***	0.068	[0.142, 0.41]	0.251
Social contact	*v*	-	-	-	-	0.112**	0.038	[0.037, 0.188]	0.120
*Cross-lagged effects*									
SIU → Loneliness	*d*	−0.387**	0.146	[−0.673, −0.101]	−0.070	−0.367*	0.145	[−0.652, −0.083]	−0.076
Loneliness → SIU	*e*	−0.012	0.006	[0.024, 0.000]	−0.060	−0.011	0.006	[−0.023, 0.001]	−0.050
SIU → Contact	*a*	-	-	-	-	−0.160	0.307	[−0.762, 0.441]	−0.012
Contact → SIU	*c*	-	-	-	-	0.001	0.002	[−0.003, 0.006]	0.018
Contact → Loneliness	*b*	-	-	-	-	−0.015	0.011	[−0.037, 0.007]	−0.037
Loneliness → Contact	*f*	-	-	-	-	−0.038	0.066	[−0.168, 0.092]	−0.015
*Mediation effects*									
ODE(SIU → Loneliness)		-	-	-	-	−0.077	0.043	[−0.161, 0.008]	−0.011
OIE(SIU → Loneliness)		-	-	-	-	0.001	0.003	[−0.005, 0.007]	0.000
OTE(SIU → Loneliness)		-	-	-	-	−0.075	0.044	[−0.161, 0.011]	−0.011
ODE(Loneliness → SIU)		-	-	-	-	−0.002	0.002	[−0.005, 0.001]	−0.008
OIE(Loneliness → SIU)		-	-	-	-	0.000	0.000	[0.000, 0.000]	0.000
OTE(Loneliness → SIU)		-	-	-	-	−0.002	0.002	[−0.005, 0.001]	−0.008
*(Co)variances*									
RI(SIU)	Var(κ)	0.050***	0.004	[0.043, 0.057]	1.000	0.050***	0.004	[0.043, 0.057]	1.000
RI(Loneliness)	Var(ω)	3.001***	0.230	[2.550, 3.453]	1.000	3.017***	0.231	[2.564, 3.469]	1.000
RI(Contact)	Var(θ)	-	-	-	-	14.848***	0.857	[13.169, 16.528]	1.000
RI(SIU), RI(Loneliness)	Cov(κ, ω)	−0.043**	0.016	[−0.075, −0.012]	−0.112	−0.043**	0.016	[−0.075, −0.011]	−0.111
RI(SIU), RI(Contact)	Cov(κ, θ)	-	-	-	-	0.234***	0.036	[0.163, 0.304]	0.271
RI(Loneliness), RI(Contact)	Cov(ω, θ)	-	-	-	-	−2.289***	0.224	[−2.727, −1.850]	−0.342

Table shows estimates of unstandardized regression parameters and corresponding standard errors. SIU = Social internet use; SE = Standard error; RI = Random intercept. Associations in both models are controlled for covariates. * *p* < .05, ** *p* < .01, *** *p* < .001.

## Discussion

Although older adults increasingly engage in SIU, many older adults still question the quality of online interactions^54^. This disposition dates back to the early years of the internet^55^when online interactions were believed to displace offline interactions. The results of this study contradict this belief by demonstrating the value of online interactions in decreasing loneliness. Nevertheless, due to the relatively small associations, some caution is appropriate.

### How SIU affects loneliness, mediated by social contact

The association between SIU and loneliness over time is consistent with previous longitudinal research^19,20,32^. Our study strengthens the existing evidence on this association by utilizing longitudinal data of loneliness and SIU and incorporating the frequency of SIU. However, the longitudinal associations were small in terms of size and significance, while the correlation coefficients between SIU and loneliness were moderately large, and the random intercepts of the RI-CLPMs were highly significant. This suggests that a large portion of the covariance between the variables can be attributed to between-person variability^49^. When the variables of interest are not measured at multiple time points, the between- and within-person variations cannot be separated. By using longitudinal and sensitive measurements of loneliness and SIU, our results show that the within-person associations between loneliness and SIU are significant and valuable but generally smaller than found in previous research. This suggests that previous cross-sectional studies might have overestimated the within-person estimates.

We found that although higher SIU is related to lower loneliness, this is not mediated by an increase in the number of close contacts with whom older adults have frequent contact, thereby contrasting previous research^20,24,32^. Social compensation^36^ provides a possible explanation for this. For older adults, it becomes increasingly more challenging to maintain contact due to functional decline^56^which can result in a decrease in in-person social contact. It could be that decreasing in-person contact is replaced by SIU. Although the net result is that contact frequency does not increase, older adults might still use SIU for strong-tie interactions.

Additionally, other mechanisms might underlie the association between SIU and loneliness. Next to relationship maintenance with strong ties, SIU also offers an easy way to connect with a large group of weaker ties and creates a feeling of belonging^57^. Lower loneliness is predicted by a combination of frequent contact with strong ties and a large number of weak ties, as weak ties offer social support and provide access to social groups^58^. While frequent contact with very close family and friends is important, older adults might primarily achieve that face-to-face, while using online contact for maintaining the broader circle of contacts.

### How loneliness affects SIU, mediated by social contact

We also hypothesized the reversed relationship between loneliness and SIU, for which we found no support. Based on the social compensation and social enhancement hypotheses and models of technology adoption, we assumed that more lonely individuals would have less opportunities for SIU. The reality might be more nuanced, in that SIU might be beneficial for some lonely older adults. Especially for those having difficulties with in-person conversations – for example due to hearing problems – SIU might be a solution due its asynchronous nature, giving more time to respond^33,59^. Future studies could specifically focus on moderators of the effect of loneliness on SIU.

Furthermore, we did not find a mediating effect of social contact. Given that other studies have shown that loneliness is strongly related to social contact, a lack of this association in our study is surprising. Furthermore, the robustness checks and post-hoc test revealed that the time interval or cut-off value for frequent contact did not change this conclusion. Loneliness is multifaceted and has a large number of risk factors and consequences^60,61^. Furthermore, the De Jong Gierveld Loneliness Scale considers both emotional and social loneliness^47^ covering different aspects of the social network. It might be that the number of frequently contacted close ties might primarily evaluate emotional loneliness – the overall scale score might be affected by other factors relating to other aspects of loneliness, such that they mask an association between loneliness and contact frequency.

Relating to the previous point, gerontological research has shown that older adults, compared to younger adults, increasingly focus on contact with a smaller group of strong ties^62,63^. It might therefore be the case that loneliness not necessarily relates to a lack in the number of frequently contacted strong ties, but rather in the contact frequency with strong ties in general, or in the lack of a few emotionally important ties. We deliberately chose the number of contacts as a mediator, both for theoretical and methodological reasons, but future studies could assess, for instance, average contact frequency with network members as a mediator.

We should consider that the measurement at T4 was taken during the COVID-19 pandemic. The pandemic increased both older adults’ SIU and loneliness^64,65^ and as in-person contact was restricted involuntarily, the relationships between only contact as substitute for in-person contact and loneliness might be different during the pandemic. However, longitudinal research performed during the pandemic shows positive cross-lagged associations between frequency of SIU and subsequent face-to-face contact^66^ and negative associations between SIU and loneliness^15^ indicating an enhancement rather than a substitution. Judging from the model comparisons, the model with restricted regression parameters and grand means fitted the data well, indicating at least a relative stability of associations over time. As the T4 measurement was taken in 2021 and 2022, it could be that the initial reaction to the restrictions in terms of SIU and loneliness was over^67^. Older adults may have found a way to cope with the situation, either by finding other means of communication or by adjusting expectations, resulting in a relatively small effect of the pandemic on our results.

### Limitations

Some limitations of this study should be taken into consideration. Our measure of SIU specifically asks for social uses of the internet and includes frequency of use. However, the questions about different internet activities are preceded by specific questions about the frequency of mobile phone use for instant messaging. Participants might have excluded their mobile phone use when answering SIU questions, thereby possibly underestimating their SIU and the resulting associations.

Related to the previous point, our measure of SIU does not distinguish between different forms of SIU, such as WhatsApp and Facebook. To fully understand the potential of SIU in decreasing loneliness, future studies should focus on this difference. Previous literature in younger adults has revealed that different types of social media have varying effects on loneliness^68^ WhatsApp is used more for deep conversations with close ties than social media^69^ so this potentially has the largest effect on older adults’ loneliness.

In the measurement of contact frequency with network members, respondents are asked to think about more than just in-person contact and also include, for instance, phone calls or writing. While specifically asking for a broader range of contact opportunities, SIU is not mentioned explicitly. Therefore, we cannot be certain that all respondents equally included SIU in their definition of contact frequency – likely, this differs across respondents.

Concerning our covariates, due to computational issues with model convergence, it was not possible to model time-variant values of covariates such as partner, work, and health status. Instead, we modeled their value on T1 on all observed variables, while freely estimating the coefficients. Life events such as retirement, health decline, or partner loss, can have a large effect on both loneliness and SIU, so the time-specific influence of these covariates should be considered in future studies with larger samples, using less computationally demanding models, or by specifically looking at the changes around one specific life event.

Furthermore, while cross-lagged models allow to assess the plausibility of different causal pathways, they cannot confirm causal orders using observational data. Our results strengthen existing evidence on a beneficial relationship between SIU and loneliness, now including specific measures of (frequency of) SIU. Note, however, that the standardized effect was rather small (b = 0.070), meaning that there are other relevant variables that explain variations in loneliness and SIU.

Lastly, the LASA was designed to be representative for the Dutch older population with their initial sample selection and the refresher cohorts. However, inherent to longitudinal cohort studies in older adults, there is a response bias towards relatively healthy respondents due to attrition over time.

## Conclusion

Using longitudinal panel data of Dutch older adults, we have shown that increased social internet use is associated with a decrease in loneliness over time. We did not find evidence of mediation of social contact. Conversely, increased loneliness was not associated with lower social internet use over time, nor was there a mediating association of social contact. These results strengthen existing evidence on a beneficial relationship between digital contact and loneliness, and warrant further development and evaluation of digital interventions to decrease loneliness in older adults.

## Data Availability

Data from the Longitudinal Aging Study Amsterdam (LASA) are available for use for specific research questions, provided that an agreement is made up. Research proposals should be submitted to the LASA Steering Group, using a standard analysis proposal form that can be obtained from the LASA website: www.lasa-vu.nl. Files with data published in this publication are freely available for replication purposes and can be obtained using the same analysis proposal form. The LASA Steering Group will review all requests for data to ensure that proposals for the use of LASA data do not violate privacy regulations and are in keeping with informed consent that is provided by all LASA participants.
